# Driving Mechanism of Power Battery Recycling Systems in Companies

**DOI:** 10.3390/ijerph17218204

**Published:** 2020-11-06

**Authors:** Baojian Zhang, Jianqiang Li, Xiaohang Yue

**Affiliations:** 1 School of Management Science and Engineering, Shanxi University of Finance and Economics, Taiyuan 030000, China; zhangbaojian190@163.com; 2 School of Business, University of Wisconsin-Milwaukee, Milwaukee, WI 53211, USA; xyue@uwm.edu

**Keywords:** battery recycling, technological innovation, alternatives, system dynamics

## Abstract

In recent years, international environmental and public health research has become a hot topic, and battery recycling, which is often mentioned separately from waste disposal, has likewise become an academic topic. Battery recycling research is beneficial not only for controlling toxic and harmful substances, but also for public health. In addition, battery recycling brings value-added benefits to company management. As the most important link in the battery supply chain, the driving mechanism of battery recycling in the new electric vehicle industry will become particularly important. The subject of battery recycling is diverse, and the relationships among influencing factors are complex, thereby presenting a fluctuating state. Against this background, this study constructs a system dynamics model from the perspective of a main sorting and recycling system, a technological innovation subsystem and a replacement subsystem. Moreover, this study examines the driving mechanism of the power battery recycling system of a microlevel company. Focusing on the systematic impact of technological innovation capability and substitution, we find that the technological innovation drive of companies increases the total effect of required costs and product demands. It is embodied in two aspects, that is, the increase in the recovery rate leads to an increase in demand, whereas the increase in actual expenditures is less than the increase in technology-driven benefits. After technological innovation capability is improved, the effect of the technological innovation multiplier on the driving mechanism of companies is shown as rapid response time changes. In the substitution component of a company recycling system, we find that the maximum substitution rate limiting expectations has no significant impact on product differentiation. The leading effect of technological innovation capability is more obvious than that of substitution activity. Based on these findings, several suggestions for company operation and environmental governance are presented.

## 1. Introduction

China has paid increasing attention to ecological and environmental protection and public health. Currently, guided by the conviction that lucid waters and lush mountains are invaluable assets, China is advocating the harmonious coexistence of people and nature and maintaining its path towards green and sustainable development. The problem of waste classification is becoming increasingly prevalent not only in China, but also across the world. The problem of environmental pollution and nonrenewable energy depletion has likewise become serious. As toxic and harmful waste, batteries should be given considerable attention. Driven by power batteries, electric vehicles can reduce carbon emissions from the transportation industry and people’s dependence on oil, as well as promote the development of the automobile industry chain. Therefore, vigorously developing new-energy vehicles is an important measure for developing a low-carbon economy [1]. Battery power system technology, as a key factor restricting electric vehicles [2], is constantly improving under the influence of the economy and environment in the direction of small volumes, long life cycles and high safety performance. In China, according to the specified warranty period of power batteries of eight years or 120,000 km, the first batch of power batteries was eliminated on a large scale based on the 2012 energy saving and new-energy vehicle demonstration and promotion plan. The average annual growth rate of new vehicle sales in the past 10 years had exceeded 24% [3]. Such development and promotion has caused more and more pressure on battery recycling of new energy vehicles. Waste power batteries contain strong corrosive, poisonous and harmful substances, and heavy metals. Power batteries contain heavy metals, such as Nickel and carbon monoxide, as well as electrolytes, which are harmful to the environment. If not handled properly, waste batteries can pollute the atmosphere, soil, and water. Hence, the recycling of waste batteries is of considerable importance to the ecological environment and public health. In the centralised recovery of batteries, treatment will improve not only effectiveness but also the enthusiasm of people to recycle used batteries [4].

In this study, we establish a system dynamics model by investigating and analysing the recovery system. The establishment of the model is based on the optimisation and driving mechanism of the recycling and reprocessing process of waste power batteries. We use life cycle assessment (LCA) in the modelling process, which is a type of systematic tool for assessing environmental impacts associated with products or service systems, considering upstream and downstream activities. Moreover, LCA is often used in design engineering and manufacturing [5], environmental assessments [6], and other aspects.

This study also integrates demand differentiation, demand elasticity and other factors into the model. At the same time, the economic performance and environmental performance of companies are affected by active environmental behaviour and innovative technology. Based on this idea, a dynamic model for the power battery recovery system of a corresponding company is established and analysed. In addition, the boundary of the system, namely, the research object, and its scope represent the increase and decrease of the amount of battery recycling outside a company, the change range of substitutes and changes in the driving factors of a company, such as technological innovation, demand change, the sorting multiplier, and so on.

Research on the driving problem of power battery recovery in companies can solve the specific change trend of individual influencing factors under the multiple influences of product recovery rate, product demand, actual cost, product differentiation, product substitutes and other factors under the capability of technological innovation. What is the specific driving role of technological innovation? In the recycling system, is technology or alternative intervention dominant? What types of measures will exert a substantial effect on a company? Through simulation and deduction, the model can help the essence of the internal drive and improve company efficiency. At the same time, the model can improve companies’ sense of social responsibility and increase their responsibility for the environment and social public health.

## 2. Literature Review

### 2.1. Recovery Mode

In the existing literature, most studies on the recycling of waste power batteries of new-energy vehicles focus on the issue of the recycling mode. Firstly, on the establishment of the recycling network mechanism, the scholar Schultmann established a recycling system for the waste battery hybrid closed-loop supply chain, which combines the optimisation model of reverse recycling network planning with the process simulation model of customised selection for the potential recycling of waste batteries. The results showed that waste batteries can be recycled by optimising the existing recycling structure [7]. Ene proposed a mixed integer linear programming model to study the multicycle, multilevel, and capacity-constrained network design of the reverse logistics of end-of-life vehicles [8].

In terms of recycling mode selection, Savaskan et al. examined manufacturers’ reverse recycling channel selection and proposed three options for selecting the appropriate reverse channel structure for recycling waste products from consumers, that is, recycle waste products directly from consumers, provide appropriate incentive mechanisms to induce retailers to recycle waste products and outsource waste products to a third party collection and recycling company [9]. Scholars likewise focused on the selection of product recycling mode channels. Sodhi and Reimer used bulk recycling, disassembly recycling and smelter recycling. Based on this recycling model, specific mathematical functions were developed to explain the battery recycling problem [10]. Through a game model, Xiong and Liang considered consumers’ environmental awareness factors and established a recycling decision model for manufacturer recycling, manufacturer-commissioned retailer recycling and manufacturer third-party recyclers recycling [11]. For an automobile group, Zhu and Chen established an evaluation index system to investigate the waste power batteries produced by electric vehicles and used the fuzzy comprehensive evaluation method to examine the reverse logistics mode selection of waste power batteries [12].

However, limitations exist in the optimisation of the recovery mode, and continuously improving the battery recovery system is impossible.

### 2.2. Recycle Body and Incentives

The article about the main subject of waste power battery recycling clearly defines the important role of each responsible subject in the recycling mechanism. 

The recycling model involves the creation of responsibility for a recycling subject. Numerous scholars conducted quantitative analyses on regulations on producer responsibility systems for recycling subjects. Shi divided responsibility sharing into three reverse channels, that is, retailer recycling, manufacturer recycling and third-party recycling, then compared the three models with a situation not considering responsibility sharing and analysed and quantified responsibility sharing as enjoyment validity [13]. Subramanian examined the effect of extended producer responsibility system parameters on durable product designs and the supply chain coordination incentive mechanism [14]. Toktay designed a cost allocation mechanism that allocates part of the production cost to a remanufacturing department [15]. Atasu and Subramanian compared the impact of collective producer responsibility and single producer responsibility on the design and return of manufacturers’ reusable products and consumer surplus. The authors believed that the single mode generates more incentives than the collective mode in promoting manufacturers’ design of reusable products and that collective producer responsibility could produce high consumer surplus [16]. In China, according to national regulations, new-energy vehicle companies are the main body responsible for the recovery of waste power batteries. In addition, manufacturers establish battery recovery outlets according to such standards. After waste power battery recovery, recycling outlets return the batteries to the companies. Once preliminary identification is complete, the companies will transfer the batteries to either locally designated echelon utilisation companies or recycling companies depending on the waste and old batteries’ degree of decay and loss. The used battery recycling supply and important role of new-energy vehicle companies in the production and recovery of used batteries have increased. Furthermore, the significant role of research on companies’ recovery drive for power batteries has likewise increased.

Incentive measures for the mechanism of the recovery system are the key factors driving the improved operation of the recovery mechanism. Related articles also combined recycling modes and policy effects with recycling pricing to determine recycling incentives.

Using a cooperative game model, Vimal Kumar Gupta et al. reviewed and critically analysed the applicability of a stakeholder policy in an alliance framework. The results showed that manufacturers play a leading role in recycling and that waste management is considerably influenced by producer responsibility. Therefore, in recycling, we deal mainly with manufacturers [17]. Thus, the problem of new-energy vehicle recycling should be resolved from the battery design source and manufacturing.

In terms of the effect of policy subsidies, Mitra and Webster assessed the impact of different recycling legislations on the interests of members of the competitive environment of new and remanufactured products and found that the effect of a sharing subsidy policy is superior [18]. Govindan analysed the coordination mode of a two-level closed-loop supply chain in the recycling mode, showed that using contracts to coordinate a closed-loop supply chain policy is effective and classified and compared the advantages and disadvantages of various ways [19]. Based on this conclusion and the life characteristics of power batteries, Xie analysed a waste power battery business model and found that according to an evaluation of a future policy development trend, power batteries will be bound to electric vehicles through coding, and consumers will take the initiative to deliver waste power batteries to power battery treatment units. This process will realise the effective recovery of power batteries in the entire life cycle of electric vehicles. This conclusion can provide a satisfactory theoretical basis for battery recovery drive in the entire life cycle [20].

### 2.3. Driving Factors and Business Operations

The influencing factors of waste power battery recycling are key to the development of a company. Scholars used different methods to analyse the impact of various product factors on companies and the environment. Through electric vehicle battery recovery mathematical modelling and simulation, Liu and Gong analysed factors affecting battery recovery and the degree of influence of the factors. The authors believed that impact on recycling depends largely on the relative life [21]. Wen et al. also found that recovery plays a crucial role in the recovery of electronic products [22]. To optimise the total profit of the entire supply chain in different battery life cycles, Gu and Petros presented an optimal pricing strategy between manufacturers and remanufacturers and discussed the relationship between recovery rate, sorting rate and recovery rate. The results showed that the recovery and reuse of batteries can help reduce raw material consumption and impact on the environment [23]. Schaik and Reuter examined the impact of battery recycling on the environment based on product design and recycling technology using the principle of system dynamics. Based on recovery rate and battery life, the improvement of production technology capacity plays a decisive role in the development of a company [24]. Su Jing found that technology orientation has a significant impact on the performance of startups in his study on technology orientation, and the substitution capability of substitutes can regulate the relationship between them [25]. Waste power batteries are affected by various factors and are key to the internal recovery of companies.

Technological innovation is indispensable in the development of companies. In the study of Sarah King and Naomi, Australia promoted technology development and innovation to support emerging industry. The authors considered the expected growth in consumption and demand for portable equipment and electronic vehicles, the potential economic benefits of lithium-ion battery materials, the lack of infrastructure and capacity to deal with wastes, and the development of policies and regulations for their management [26]. This situation is used worldwide. Innovation, as an inexhaustible source of company development, is the main wing of company recovery systems. However, in the combination of technological innovation and recycling influencing factors, such as innovation and alternative research, how to adjust recycling and reprocessing companies’ driving force is an interesting topic under the overall recycling system. 

However, most studies on the input supply chain structure are static analyses and do not reflect the passive battery consumption situation. Conducting research on a recycling subject and the incentives of an enterprise strategy is difficult. As a dynamic company behaviour with multiple subjects, diverse influencing factors and complex relationships, battery recycling must adopt the system dynamics method to accurately reflect the specific battery recycling process. System dynamics is often used in business decision making, waste management, industrial engineering management and other fields. It can effectively address the effect of policy implementation and the nonlinear structure of realistic assumptions. Moreover, system dynamics can understand the complex relationships among different elements from a holistic perspective, predict dynamic feedback from historical dimensions and make predictions using model boundary, structure, and parameter adjustments to optimise a model. From the overall perspective of a company, the system dynamics research method is an effective way to resolve the system driving problem as a whole. Previously, scholars used system dynamics to study the recycling of waste power batteries. Hou constructed a system dynamics model for added electric vehicle amounts based on an analysis on the relationship between power batteries and electric vehicles. The author predicted the addition of electric vehicles in the future and calculated the new power battery increment [27]. Wang Lili analysed the internal recycling and remanufacturing behaviours of companies in the used battery reverse supply chain system and employed system dynamics and cognitive behaviour theory based on planning behaviour theory to examine the impact of relevant social factors on consumers’ acceptance of remanufactured batteries. In addition, the author analyzes it from the perspective of consumer behaviour and drew on the strategies of companies’ recycling and remanufacturing capacity in different situations. Meanwhile, the government and battery companies should encourage and guide consumers to use remanufactured batteries in the early stage [28]. However, the present study focuses on choices from the consumer side and does not comprehensively examine the overall perspective of a company. With technological innovation creating recycling innovation and product replacements, the power battery processing and reuse process has improved. Companies’ recycling and reprocessing of waste power batteries involve a complex, dynamic, and nonlinear process. Moreover, revealing the internal laws and influences of factor changes on the overall model using a conventional analysis method is difficult. Based on this assessment, this study uses the system dynamics method to analyse these complex processes and their impact and department system internal factor mechanism operations.

## 3. Model Building

From the perspective of companies, we use the system dynamics research method with the software of Vensim PLE (Ventana Systems Inc., Harvard, MA, USA) through a comprehensive analysis of the literature and theory of planned behaviour in marketing to discuss the dimensions of the recycling end of waste batteries.

We begin with an analysis of industry development reports and the annual reports of some major companies. Then we conducted three specific field investigations on new energy vehicle companies. Finally, we select a typical company that can reflect the industry standard level for detailed data analysis and assignment for model analysis.

In addition, we analyse control changes in companies at each end of the battery recycling subject. Theory of planned behaviour and the general decision-making process of individuals or companies are assessed from the perspective of information processing. Making decisions based on the competition of substitutes of external environment factors to conduct information processing, production and marketing is a process of continuous improvement. Based on a follow-up investigation of the waste power battery recovery system of a new-energy vehicle company, we construct a system causality diagram and employ the relationship among system dynamics factors to construct an equation. We complete the dynamic causality characterisation in the flow diagram, then analyse the dynamic simulation by testing the key factors.

The model construction steps are described below:

Causality diagram: According to operations, companies determine the characteristics of each link through a company recycling improvement process follow-up inspection. In this study, a causality diagram is drawn based on an analysis and research of the variables needed in the specific research. Based on previous research experience and the specific situation of the driving mechanism of enterprise power battery recovery, the relationship between the main factors and variables and the promotion and inhibition effects between the variables are determined, and the corresponding causality diagram is drawn.

System flow diagram: Based on causality, the relationship between the variables is treated nonlinearly, and an equation for each variable is determined to construct the system model. The initial data used in the system flowchart in this study are based on the investigation of three companies and the in-depth data investigation of one company reflecting the average level of the industry. In the data investigation, secondary data, such as industry development reports, are employed. The establishment of the equation in the system flowchart is also based on previous experience and the specific situation of the industry.

Model testing and tuning: Consistency between the model and reality is verified. The test function is likewise verified, and the overall model structure is adjusted by testing the changing objective law and trend of the output. The model test and adjustment involve checking the validity and reliability of the model, which can ensure the simulation degree and consistency between the simulation results and reality. Specifically, there are ‘system boundary rationality test’, ‘unit consistency test’, ‘extreme test’, and ‘abnormal behaviour test’ to verify whether the results are consistent with the actual situation.

Model simulation: The PLE software Vensim is used to adjust the variables and observe the trend of the target variables. In the model simulation, the parameters and initial variables are adjusted accordingly based on the simulation result analysis, and changes in the target variables are observed by increasing or decreasing the variables to directly or indirectly observe the driving mechanism of company battery recovery.

Suggestions: Based on the results of the simulation analysis, corresponding policy recommendations are presented. Through research on the driving mechanism, this study provides suggestions on the operation driving relevant companies. Meanwhile, owing to the particularity of the research object, this study can provide effective suggestions for battery recycling and utilisation.

**Figure 1:**
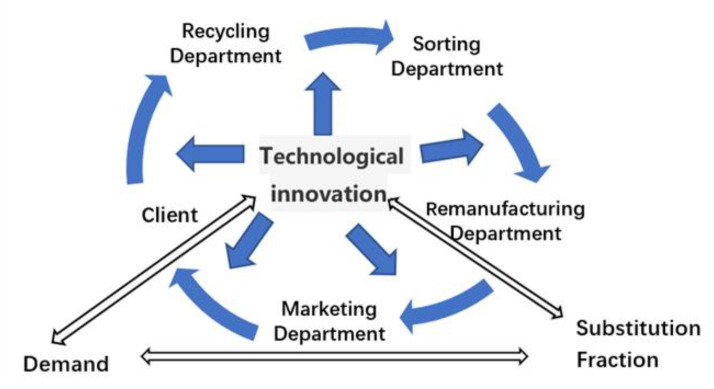
Flow of battery recovery system.

Specifically, this study constructs the supply flow of a company’s internal battery recycling system, which is presented in Figure 1.

A company recovery system is a complex and continuous abstract model. In the establishment of a system dynamics model, making assumptions and providing explanations about the relationship between the variables in the system model are necessary. In this study, the following assumptions are made for the problems that must be addressed in establishing the system model: 

Scenario 1: Only one reuse behaviour exists, namely, remanufacturing. Remanufacturing means to decompose, repair and replace returned products to enhance their quality to reach or exceed that of new products. After a remanufactured battery is complete, it is transferred to an inventory centre and sold once again to meet demands. That some batteries cannot be recycled and must be disposed of directly is not considered, as the direct disposal of waste batteries causes serious environmental pollution. According to the technical policy for the prevention and control of waste battery pollution issued in China in 2003, battery manufacturers must take responsibility for recycling waste power batteries. Therefore, all waste batteries are assumed to be recyclable. However, in the process of recycling, some materials do not meet remanufacturing standards; thus, in this aspect, this study adopts direct disposal. Therefore, the following scenario is made that under the investment of technological innovation, the product performance is bound to improve and be accepted by consumers.

Scenario 2: The recycling of used batteries is a continuous dynamic process and a long-term behaviour. Technological innovation investment acts on the entire recovery system and refers to not only the production links of products but also product disassembly, sorting and other processes. Investment in technological innovation is not a direct one-off investment but a continuous investment process within a certain time range. In the process of system dynamic driving, moderate changes in various stocks and variables need time lags to produce expected effects, which are reflected in the long-term process. As a result, it also conforms to system dynamics characteristics for examining long-term and complex network relations. Therefore, scenario 2 was established based on showing the finiteness of conditional input.

Scenario 3: After waste battery reprocessing and reuse, product performance is improved, which affects consumer behaviour. Waste battery processing and reuse are conducted in companies. The different waste battery postprocessing techniques can nearly meet the requirements of original batteries or demonstrate improved battery performance owing to technological progress. Planning behaviour theory proposed by Ajzen explains individuals’ general decision-making process from the perspective of information processing, which mainly includes five levels, namely, attitude (which directly determines consumer behaviour) [29], subjective norms, perceptual behaviour control, professional knowledge and convenience. In the long-term process, owing to product performance improvement, providing consumers with adequate stimulation and satisfactory experiences at the cognitive level can affect their attitude, thereby influencing their behaviour [30]. Therefore, based on practical considerations, we made scenario 3: battery recycling of new energy vehicles is a long-term dynamic process.

Scenario 4: Investments in human, financial and material resources are limited. Neubauer and Pesaran concluded that a portion of the cost of electric vehicles could be offset by reusing used power batteries [31]. The essence of a company is to generate profits. The recycling of used batteries can not only save resources and protect the environment but also save money. However, a company must generate profits. Thus, investment amounts are limited. Therefore, boundary values exist in the system for investment in technological innovation and the amount of capital investment. Specific boundary values are estimated according to actual situations.

Scenario 5: Each behaviour of a company is considered as an overall behaviour, and the outsourcing behaviour in each process is considered as the internal behaviour of the company. The research in this paper is the driving mechanism of waste battery recycling in companies. The system we established is an integral system within the company, so based on this reason, we set up scenario 5.

In reality, companies’ battery manufacturing may be outsourced but can be regarded as an internal behaviour. The ultimate benefit is the recycling of used batteries and the use effect of batteries as a service, which do not influence the driving effect of various factors in the recycling system on the overall operation of a company.

### 3.1. Causality Analysis of Power Batteries in Companies

**Figure 2:**
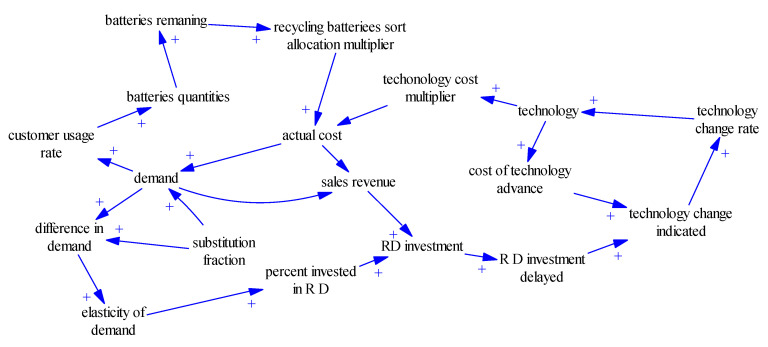
Cause and effect diagram.

Based on the previous model assumptions for specific situations, we analyse the causal relationship in the recovery of power batteries of companies. The subject of this study is the optimisation and driving mechanism in the process of the recycling, processing, and reprocessing of waste power batteries in a company and the development of a company operation system. The boundary of the study involves relevant entities and important variables in the business process. By investigating and analysing the recycling system, specifically, in company recycling departments, sorting and processing departments, manufacturing and production departments and marketing departments and customers under this closed-loop system, firstly, we determine the influence of battery substitutes on the system. Customers’ diverse demands and substitutes’ attractiveness are factors that companies cannot control directly, whereas controllable factors include customer satisfaction, customer brand preference and customers’ perceived barriers to change [32]. At the same time, the economics of company and environmental performance is influenced by active environmental behaviours and innovative technology. To optimise companies’ economic and environmental benefits, firstly, we must ensure that companies engage in scientific, rational, and active positive environmental behaviours and have a wide range of technological innovation. The embodiment of companies’ benefits experiences cycle delays and the scale economy; thus, the corresponding active environmental behaviours and technological innovation also possess such properties. Therefore, technological innovation research support from different sectors can effectively improve companies’ economic and environmental performance. Fund support can also directly improve the efficiency and economic benefits of the system. Thus, through the adoption of the company power battery recycling system to identify indicators among the various factors as well as causal relationships, a causal graph is drawn as follows in Figure 2.

The causal loop of the sorting recovery system is as follows:

Customer usage rate → battery quantity → batteries remaining → recycling battery sort allocation multiplier → actual cost → demand customer usage rate.

The technological innovation input subsystem loop is as follows:

Technology change rate → technology → technology cost multiplier → actual cost → sales revenue → R&D investment → R&D investment delayed → technology change indicated → technology change rate.

**Figure 3:**
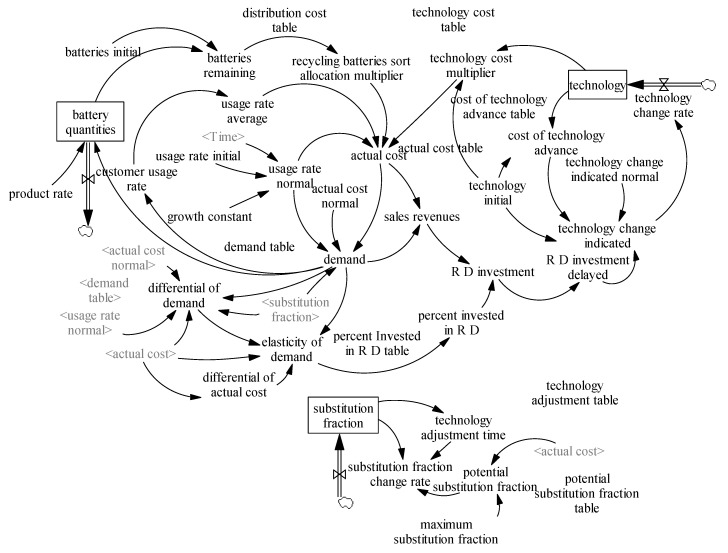
Stock flow diagram.

The stock flow diagram is determined based on the cause and effect diagram of the waste battery recycling operation system of a company. Figure 3 is presented based on the company operating system cycle structure process.

### 3.2. Establishment of Important Equations and Interpretation of Variables

In system dynamics, the linear and nonlinear relationships among variables and the interpretation of individual variables are important for building the system dynamics model. In this study, the establishment of important equations and interpretation of the variables are shown in Table 1.

## 4. Model Simulation and Analysis

### 4.1. Vensim Model Test and Verification

The purpose of model testing is to test the simulation effect of the model on the real world. By setting a specific variable as a test function, we can judge the actual analysis and prediction ability of the model. The simulation time unit of this model is set to month, the testing time is 60 months and the testing step is one month. The relatively important initial values in the setup model are shown in Table 2.

Owing to the particularity of the research object, the actual data used in this study set the initial value to be as close as possible to reality.

After the model design and equation establishment, the validity and reliability of the model are verified to ensure the model simulation and simulation results. Through a ‘system boundary rationality test’, ‘unit conformance test’, ‘extreme case test’ and ‘behaviour abnormality test’, we determine that the results are consistent with the actual situation, and the simulation results and corresponding discussions and explanations are within reasonable limits; thus, the model meets the requirements of reliability and validity. The test results of the model are collated, as shown in Table 3.

**Figure 4:**
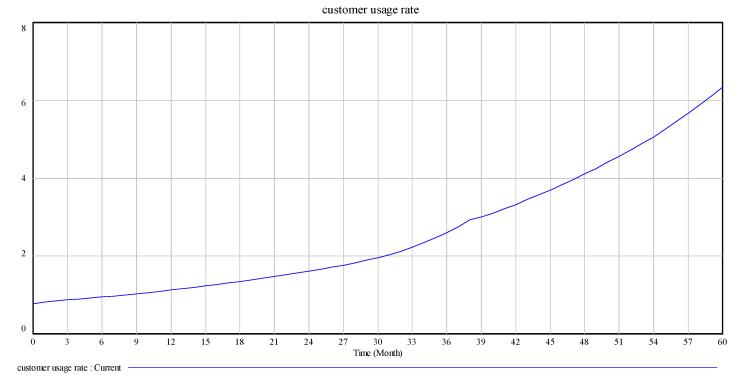
Customer usage rate.

**Figure 5:**
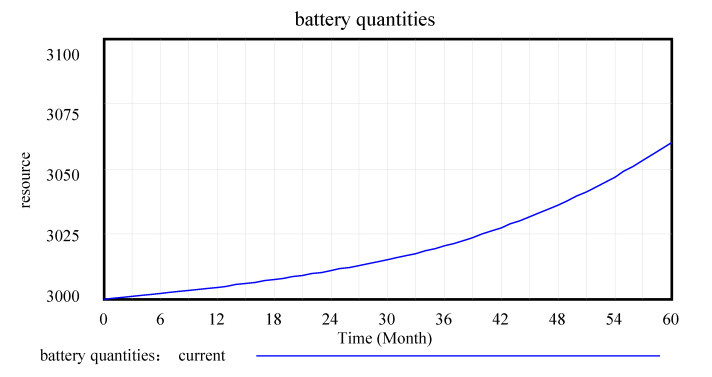
Battery quantity.

**Figure 6:**
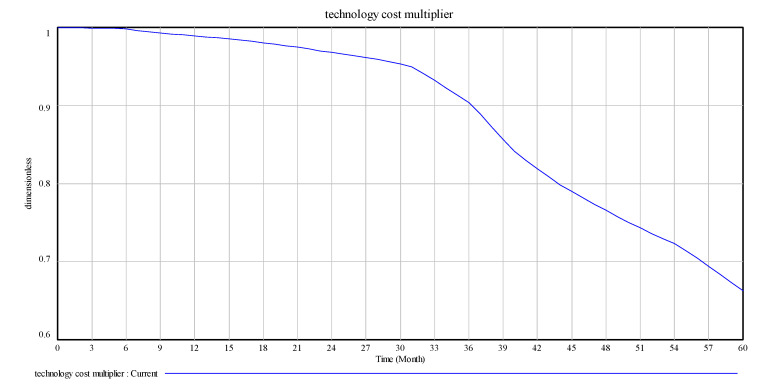
Technology cost multiplier.

After the model test and inspection, changes in the main variables of the model operation are observed, as shown in Figure 4, Figure 5 and Figure 6.

Figure 4, Figure 5 and Figure 6 are the intuitive responses to the basic variables and the variables we mainly observe, that is, the intuitive result presentation of the simulation results. Technology change indicated normal is 0.1, and the maximum substitution fraction is 0.6 under the condition of initial value technological innovation investment and alternative parameter control, considering that in 60 simulation cycles, the number and recovery rate of spent power batteries are significantly increased by the company recovery system from the initial 3000 units to over 3060 units. This result is in line with the improvement of technological innovation in the development of companies, which inevitably anticipates the increase in the number of batteries. At the same time, the consumer consumption rate for power battery products also increases significantly, and a certain degree of fluctuation in the set cycle is observed, which is in line with the expectation of innovation technology investment for the promotion of a company. This is a good driving force for new energy battery recycling enterprises in the current big environment, such as the substantial reduction in profits, lack of core technology, excess capacity, and recycling difficulties [33].

The sorting multiplier refers to the ratio of the impact multiplier to cost after used battery recycling.

The technology cost multiplier is the ratio of the impact index to cost after technological innovation investment. In this study, owing to the small increase and actual effect of the lookup function, we determine that the proportion of the effect of the sorting coefficient on actual cost tends to be stable. The technology innovation cost multiplier must carry on the investment to become large in the early stage of initial investment. The influence multiplier reduces the proportion to become small, along with the testing period number increase, in the medium-term stage of technological investment, which tends to stabilise the proportion of the impact of the increased decline trend. The influence of multiplier ratio is the quantified embodiment of the driving relationship, which has a direct effect.

### 4.2. Analysis of Simulation Results

#### 4.2.1. Analysis of Product Technological Innovation Capability Change

**Figure 7:**
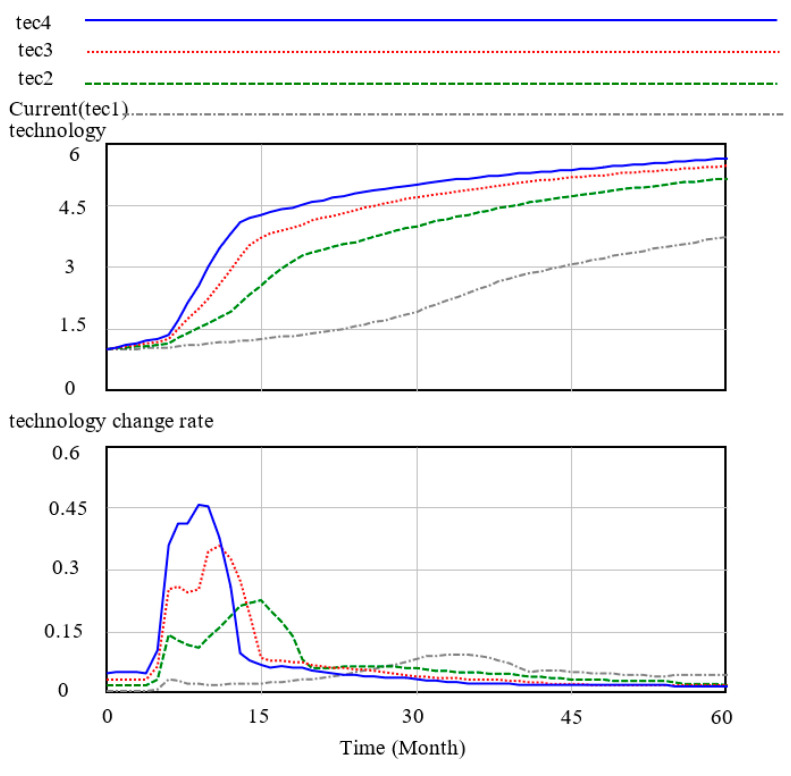
Technology and technology change rate.

**Figure 8:**
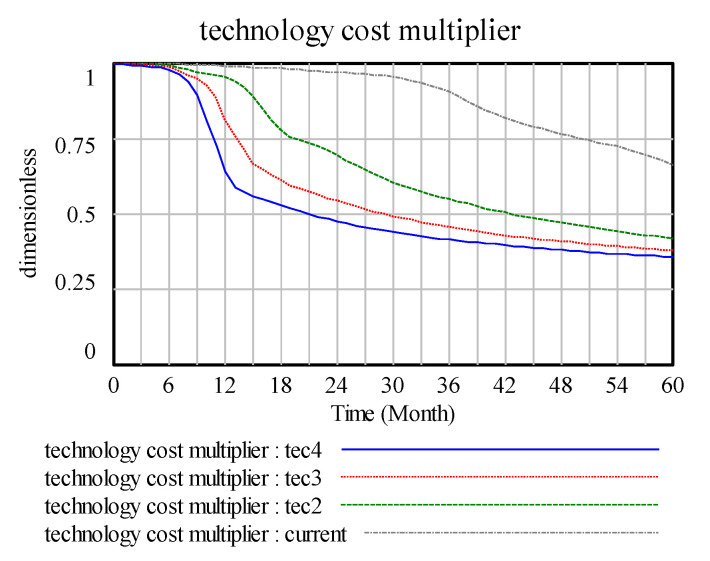
Technology cost multiplier.

In the case of technological innovation, changes in the technological innovation input increment in the technology stock and in the technological innovation rate are obvious, as shown in Figure 7 and Figure 8. At the starting value, the current technology change indicated normal is 0.1, the total trend of the increase in the technology stock is five months and the period of the increase is nearly one year. Moreover, the increase tends to be smooth, the trend of the technology change rate confirms the change in the technology stock and the peak period of the change rate is half a year after the technology change.

The Figure 7 represent current(tec1), tec2, tec3, and tec4 corresponding to 0.1, 0.4, 0.7, and 1 technology change indicated normal, respectively. Changes in technology change indicated normal mainly generate the peak value of the technology change rate, and the higher the peak value, the higher the change rate. In Figure 8, the change in the technology impact multiplier is a high technology change indicated normal, and the effect on the technology multiplier is rapid and obvious. However, a low technology change indicated normal is a slow process and takes a long time to accumulate the effect of technological input on the final cost.

#### 4.2.2. Impact of Alternatives on the Corporate Recycling System

To examine competition, we include the alternatives in the model against the background of technological innovation, considering the relationship between the growth and decline of the proportion of the alternatives and companies’ power battery recycling system. We also analyse the internal mechanism. Alternatives are a threat to business but may also present opportunities. If a company has a strong innovation capacity, then it will take the lead in introducing new cost-effective products and stay ahead of the competition. At the same time, consumers’ requirements for products continue to increase. For waste power battery manufacturers, the different qualities, prices, performances, and environmental protection of every type of battery will generate different feelings among consumers and psychological satisfaction to varying degrees. Therefore, companies manufacturing goods that can be substituted for one another must ensure their quality and accurately examine the quality and prices of similar goods in the market to guarantee that their products and prices can attract consumers and generate profits. In the study of commodities, if every company in the same industry adopts the same pricing method and the proportion is close, then prices will likewise be close. Although a company can reduce or avoid price competition, it can also ignore product demand elasticity changes; thus, its pricing foundation will lack flexibility, and the company can easily make the wrong decisions. Moreover, reducing the product cost is disadvantageous. Thus, this study includes differentiation, demand elasticity and other factors in the model.

In the alternative partial Vensim model, the variable constant is the maximum substitution fraction. It is driven by the effect of the maximum alternative restriction on the potential alternatives and thus on the change rate of the effect and partial stock of the alternatives and hence the main model in the differentiation of the commodity demand and the actual demand for the role.

**Figure 9:**
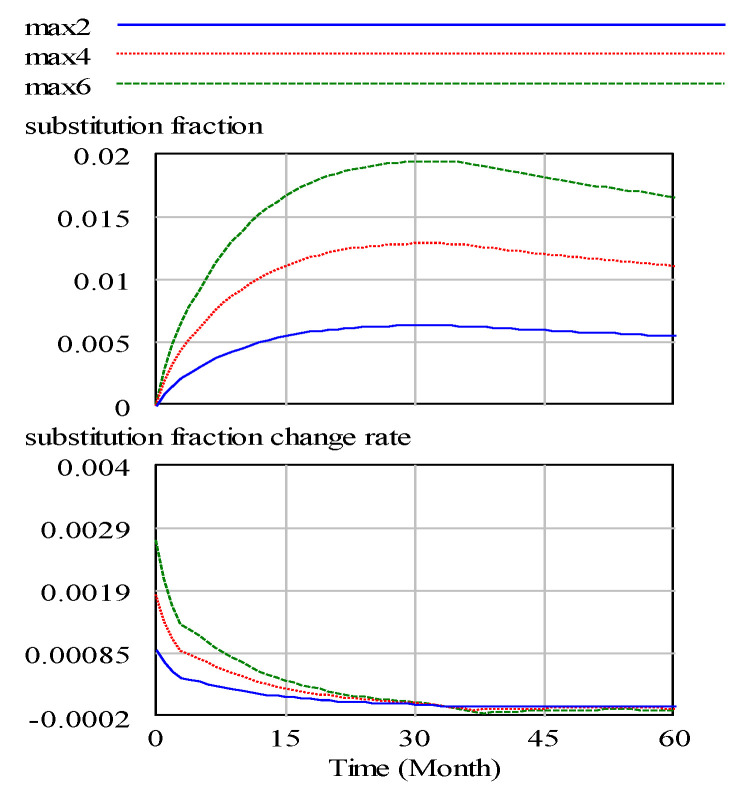
Substitution fraction and change rate. (max2, max4, max6 represent the maximum substitution fraction of the variable is equal to 0.2, 0.4, and 0.6).

**Figure 10:**
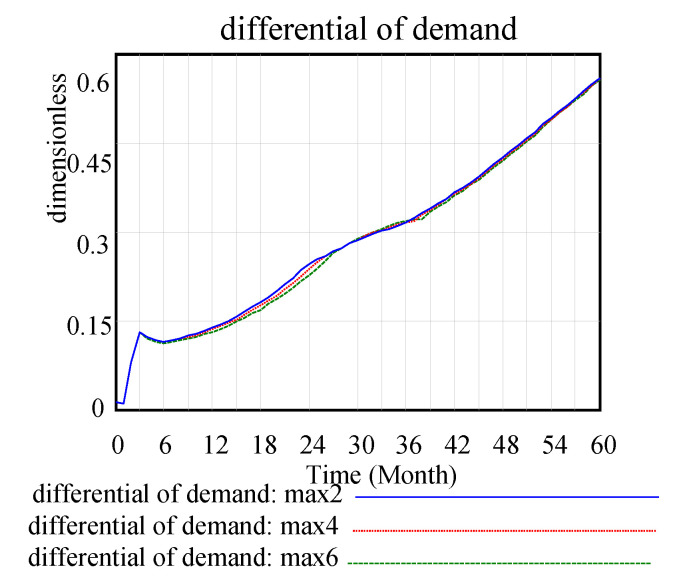
Differential demand.

In the Figure 9, max2, max4, and max6 show the partial effect on the alternatives when the maximum substitution fraction is equal to 0.2, 0.4, and 0.6, respectively. In this study, the waste power recovery system of a company examines current power battery replacement products. The system simulation determines that the proportion of the replacement is only 0.1 or less, which is in line with power batteries as a source of new energy in the current automotive industry development trend. In addition, with technological innovation investment factors, the substitution trend decreases and stabilises. With the limitation in the maximum substitution fraction, the change rate of the substitution fraction does not increase much and returns to the original level in 30-unit cycles. Thus, the maximum value of the maximum substitution fraction is limited by external means, such as technological innovation. Figure 10 shows the improvement in the maximum substitution fraction has little effect on the differentiation of the requirements.

Under the initial condition, the investment technology stock gradually increases, and the used battery quantity, demand, and utilisation rate develop to the benefit of the company.

#### 4.2.3. Impact of the Significant Change in Usage Rate

The initial usage rate is set to 0.8 in the initial setting, and the real rate value is changed when the value of the initial usage rate is changed. Given that the usage rate changes according to the value of the other variables and company benefits, we can analyse the influence of the actual usage rate variable on the other factors.

**Figure 11:**
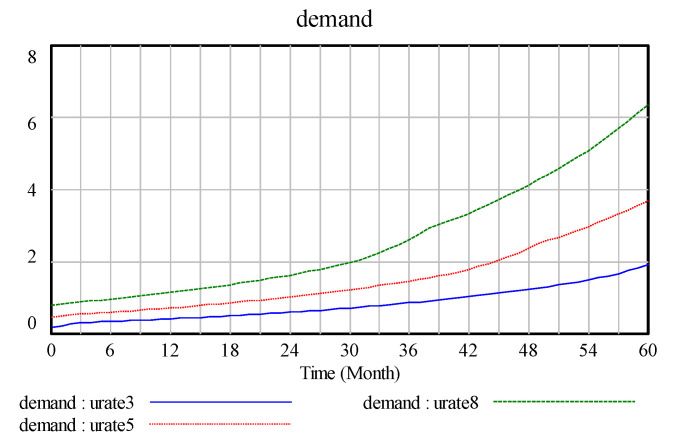
Demand.

**Figure 12:**
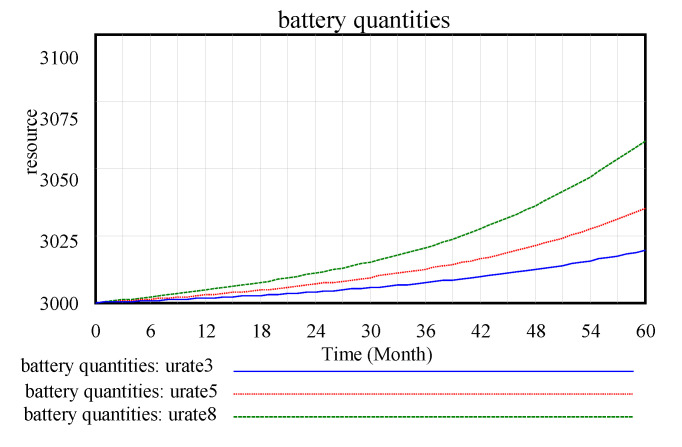
Battery quantity.

The initial usage rate is set to 0.8, 0.5, and 0.3 in this study. As the initial usage rate increases from 0.3 to 0.8, as shown in the Figure 11, in the requirement variable graphs, the increase in the usage rate leads to a significant increase in demand.As shown in Figure 12, where the increase in the number of batteries is effective, the recycling and reuse rates of waste batteries are improved and demand and company benefits are increased.

**Figure 13:**
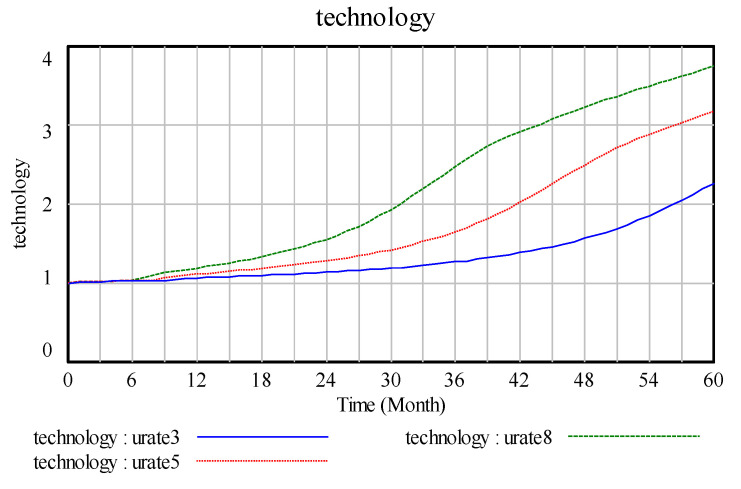
Technology.

**Figure 14:**
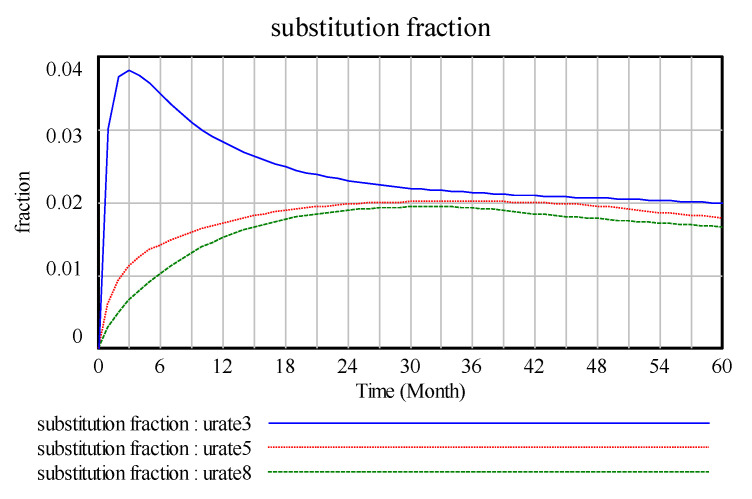
Substitution fraction.

**Figure 15:**
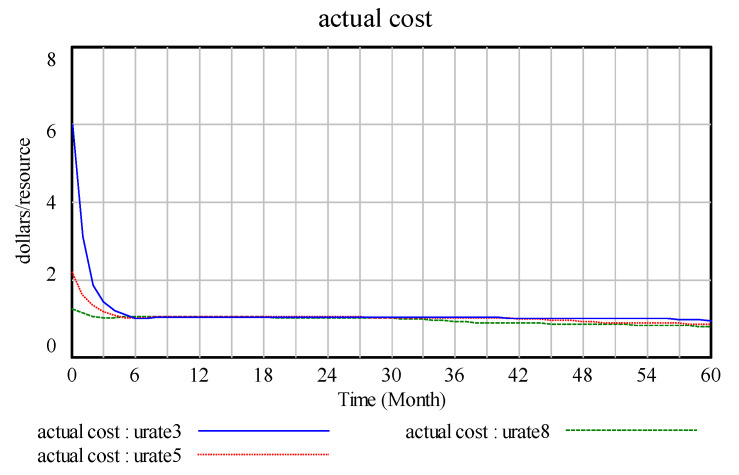
Actual cost.

Figure 13 and Figure 14 show changes in the technology stock and alternatives stock as a result of the initial change in the usage rate. Owing to the complexity and linkage of system dynamics, changes in the usage rate must be driven by technological innovation to increase demand. Under the condition of a high technology stock, the corresponding factors are a high recycling rate and a low product substitution rate. Figure 15 shows actual cost. In the battery recovery cost, the commonly believed cost includes initial investment costs, operation and maintenance costs and batteries replacement costs [34]. The factors in the system complement one another owing to technological innovation. The change in technological innovation capability has little effect on the change in actual expenditure. Moreover, the process of improving the reuse rate by improving the innovation capability of a company promotes the technological innovation, product recycling and manufacturing departments among the other systems, thereby enabling the company to experience increased product demand and gain substantial benefits.

**Figure 16:**
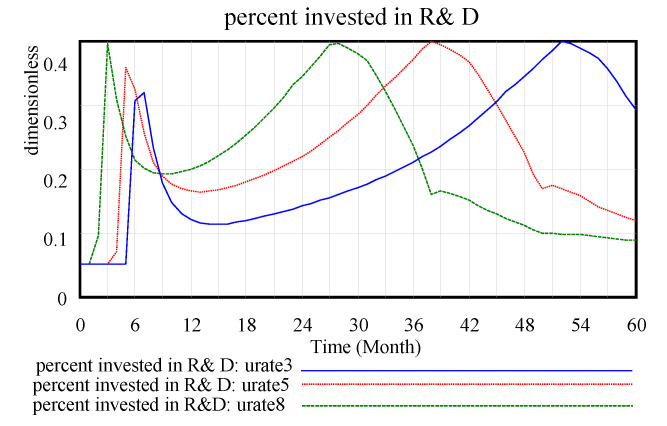
Percent invested in R&D.

In the case of the initial usage rate, the change in the rate of investment in technological innovation over the dynamic process is shown in Figure 16. As the rate changes from 0.8 to 0.5 to 0.3, the curve of percent invested in R&D moves to the right. That is, the technological innovation investment time is more delayed than expected, and this time delay indicates that a company’s response to technological innovation investment is also slow under the condition of a low recycling rate. For large usage rate images, the image duration is less than the smaller usage rate image fluctuation range. This change indicates that technological innovation investment requires less time under a high recycling utilisation rate, and a stable high rate requires a high technological innovation input rate.

### 4.3. Discussion on Model Simulation

The waste power battery recovery system of a company is a closed-loop supply chain. Battery production reaches consumers through distribution. At the end of its service life cycle, a battery returns to the company through recovery by a company recovery department or a third party. After the inspection and decomposition process of a sorting department, coupled with the drive of technological innovation, the battery is handed over to a production and remanufacturing department. Batteries that are slightly better than or similar to original batteries are sold after packaging. This model can be divided into three subsystems, that is, the sorting and recycling system, the technological innovation system, and the alternative product research system. The sorting subsystem is the most important component of a company battery recovery system, which involves a sorting multiplier for organically combining the number of batteries recovered by a company with the cost. Through a simulation, we determine that with the improvement of the recovery rate and sorting cost multiplier, the total effect of the actual cost and a company’s sales revenue recovery will move towards a direction that is beneficial to the development of the company. Specifically, the change in the growth constant will cause a change in the result variable.

In research on several substitute subsystems, they are found to be nested and related to demand elasticity and demand difference. The proportion of innovation R&D investment is determined by demand elasticity and affects the technological investment subsystem. In the substitute subsystem, the technology adjustment time, proportion of potential substitutes and proportion of maximum substitutes are introduced. In addition to performance, the cost substitute portion of a product exerts an impact on the actual product; thus, the actual cost has an important role in the potential substitute portion of the product. In the research process, we find that with the passage of time and investment in technological innovation, the potential substitutes will stabilise, thereby indicating that technological innovation investment can improve current power battery consumers.

In addition, we determine the factors affecting substitutes and that companies in different industries compete with one another owing to the similar functions of their manufactured products. This type of competition from substitutes can change the competitive strategy of existing companies to a certain extent. In an industry with a high threat of substitutes, the prices and profitability of existing products will be limited owing to the existence of substitutes that can be easily obtained by target customers. In this case, companies are inclined to reduce the cost of products to attract consumers and maintain an advantage in the competition.

In the current situation, there is a technological externality in the new energy automobile industry. It can be understood as the external diffusion of knowledge and technology, which is a positive externality. It refers to the economic behavior caused by technology imitation and sharing among competitors in the same industry. Technology externality will lead to the reluctance of manufacturers to invest in technology [35]. This is contrary to the fact that we increase technology investment in the current research to promote consumer acceptance behavior from the outside. Therefore, for current companies, the external diffusion of knowledge and technology should be strictly prevented in the process of technological innovation, so as to produce positive effect of technological innovation investment.

In our study, we find that the improvement of the technological innovation rate in the complex system dynamics model naturally reduces the technological substitution rate, changes several parameters of the substitutes, and alters the parameters of products’ technological innovation capability. In this research, we determine that changes in technology leading to company development are greater than the changes in the substitutes. The improvement of the maximum substitution fraction in the third part has little impact on demand differentiation and the other important variables of company. Changes in the fraction are caused by the prediction of the highest substitutes and that of competitors in the future.

At present, for the new energy vehicle industry that we studied, in China, the substitutes for domestic power battery products mainly include fuel engine in traditional fuel vehicles and power battery products outside China. As the component with the highest cost proportion in new energy vehicles, the power battery system directly determines the market pricing of the whole vehicle. Only when the cost of the power battery system falls to the level comparable to the engine cost of traditional fuel vehicles can pure electric vehicles have real market competitiveness in terminal price after the withdrawal of financial subsidies. At present, the cost of the power battery system of new-energy vehicles is decreasing year by year. However, it will take many years for electric vehicles to form equal competition with traditional fuel vehicles in the post-subsidy era [36]. And the cost of power battery systems abroad is also falling fast, and the threat of replacement cannot be ignored.

Therefore, when analysing the impact of substitutes, companies should focus on improving their technological innovation capability. Only by giving full play to its advantages of strong innovation and flexibility to market changes, mastering emerging technologies and leading consumer demand can it occupy a place in the competition. When the proportion of substitutes is high, uncertainty increases; thus, the environment that companies encounter becomes uncertain, and enhancing technological innovation capability is difficult regardless of whether competitors and potential substitution threats are observed.

In the research of the closed-loop technological innovation subsystem, technology change rate → technology innovation stock → technology cost maturity → actual expenditure → sales revenue → technology R&D investment → technology R&D delay → technology potential change → technology change rate. In the system dynamics model, the initial technology investment improves the technological innovation change rate; thus, technology stock investment increases significantly. In addition, owing to technological improvement, the technology cost multiplier correspondingly reduces the actual expenditure of a company and promotes its development towards an improved direction. Therefore, technological innovation capability, as the key factor driving companies’ development, must pay attention to the initial technology stock in the operation of a company and alter the change rate of technological innovation simultaneously and make timely changes according to the current technological environment to improve drive.

## 5. Conclusions

The importance attached to the recycling of waste power batteries is also attached to the current ecological environment. At present, the power batteries of new-energy electric vehicles are widely used. Companies actively promote recycling and are an important part of the normalisation of the overall recycling channel.

In this study, we establish a model based on a company closed-loop recycling system. We examine the internal driving mechanism of a company using a system dynamics model with the economic goal to reduce cost and increase income and the recovery rate. The subject of this study is a company recycling system. Through an impact analysis of technological innovation capability and the partial subsystem variables of substitutes, we explore the internal optimisation of a company recycling system and other issues. We provide the following conclusions:

(1) In a power battery recycling system of a company, the sorting subsystem is the main system, and the technological innovation and substitute components are subsystems. Through the technological innovation investment of the technological innovation system and gradual decrease of the substitute component, a company develops towards the improved direction of the total effect of cost and income.
(2) After the increase in technological innovation investment, companies’ sorting multiplier and technological innovation multiplier will tend to gradually stabilise. The effect of a high-tech innovation change rate is obvious, and the effect of a low-tech innovation change rate is slow but can achieve the expected effect.
(3) Limiting the maximum value of the maximum substitution component with external means to possess technological innovation cannot effectively change the effect of the substitution component on the model. Moreover, improvement of the prediction analysis of the replacement component of the maximum expectation has little effect on the demand differentiation.
(4) Improvement of the reuse rate under the traction of technological innovation capability has obvious effects on companies. Specifically, companies experience increased demands, but changes in actual expenditure are not obvious.
(5) In the company operation process driven by the system, the change dominated by technological innovation capability should change more than that dominated by substitutes. Only by giving full play to its advantages of strong innovation and flexibility to market changes, mastering emerging technology and leading consumer demand can it occupy a place in the competition.

In summary, in the process of company innovation, we need not worry about the cost growth of the recovery system generated by technological innovation, because the total effect is consistently increasing. When technological innovation is driven, the size of the drive is reflected in the response of the effect. In the interaction between substitutes and technological innovation, we should pay attention to technology-oriented company drive but not focus exceedingly on the intervention of substitutes. Therefore, based on the above conclusions, this study can help improve the operation of company battery systems and provide the following suggestions and insights to relevant industries. In the recycling and remanufacturing process, improved battery substitutes will gradually appear over time, which is the trend of technological development. For the main subject of companies, production should be conducted reasonably according to a demand plan to improve the total effect of cost and income. Furthermore, investment in innovative technologies should be increased. In the process of recovery, sorting, remanufacturing, production, and sales, we should pay attention to companies driven by technology. The expectation and intervention of substitute products cannot effectively affect product demands. In the final analysis, companies should save material costs, strengthen battery quality and maximise battery recycling. At the same time, we should attach considerable importance to environmental protection and make substantial contributions to the green cycle.

Automobile companies’ active promotion of recycling is an important part of the normalisation of the overall recycling channel, and their role in waste battery recycling is obvious. This study’s conclusions can provide a new index for the influencing factors of the driving mechanism of an enterprise recycling system under the enterprise environment and a reference for the impact of the combination of the effect of substitutes and technological innovation improvement on an enterprise system. Finally, the conclusions can also serve as a reference for future system optimisation and decision support and encourage enterprises to make substantial contributions to the world environment and public health.

This paper studies the driving mechanism of company waste battery recycling, and solves the problem of sustainable development of waste battery recycling from the perspective of technological innovation. However, with the consumption of electric vehicles becoming mainstream, waste battery recycling needs to be analyzed in a more long-term way from the perspective of industry. With the development of the Internet of Things and the penetration and integration of the manufacturing industry, the recycling of waste batteries combined with the research of the Internet of technology will be our future research fields.

## Figures and Tables

**Table 1:** Equations and interpretation of variables.

Equations	Interpretation
Battery quantities = (−customer usage rate) + demand × product rate	Number of batteries is based on the initial value of the consumption rate and reproduction of the combined effect
Actual cost = actual cost table × (usage rate average/usage rate normal) × recycling batteries sort allocation multiplier × technology cost multiplier	Actual cost table is a lookup function, where the combination of the sorting multiplier and technology cost multiplier affects actual cost
Technology change indicated = technology change indicated normal ×(1/cost of technology advance) × R&D investment delayed × technology initial	Technology change indicated is an important auxiliary variable in technological innovation change, which is influenced by the initial R&D investment amount, the potential variable and the cost of technology
Substitution fraction change rate = (potential substitution fraction − substitution fraction)/technology adjustment time	Partial change rate of a substitute product is an auxiliary variable of the substitute product system, which is the result of the potential replacement, the replaced part and the technological adjustment time
Usage rate normal = usage rate initial × EXP (growth constant × [Time − 0])	Usage rate involves an exponential increase in the initial rate of usage depending on the length of time
R&D investment delayed=DELAY3(R&D investment,3)	A certain time LAG EFFECT in R&D investment exists, which is assumed to be three units

**Table 2:** Initial variables.

Variable Name	Initial Value	Unit
Product rate	1.4	Product
Growth constant	0.03	Product/month
Technology initial	1	Technology
Initial processing rate	0.8	Product/month
Technology change indicated normal	0.1	Technology/month
Maximum substitution fraction	0.6	Fraction

**Table 3:** Model test results.

Test Methods	Test Items	Assumed Parameters and Specifications	Test Results
**System boundary rationality test**	Tests whether the key variables and important concepts in the system are endogenous variables; tests the sensitivity of the system to changes in the system boundary	Observes whether the system can form a complete loop by adding and subtracting variables	System can form a complete loop by adding and subtracting variables
**Unit consistency test**	Checks the consistency of all variable units in the model	Check model in Vensim is used to verify whether the model is running smoothly with consistency	Through model verification, all the variable units in the model are consistent, and the model can run smoothly
	Output values of the variables are all between assumptions	In the establishment of the equation, the variables are considered, and all variables regarding the ratio are between 0 and 1	Unit-wide consistency is reasonable
**Extreme test**	Steady state test	Adjusts some of the variables to zero	Test results agree with the general behaviour of the system
	Performance scenario analysis; effect of time prolongation	Extends the testing time of the model	Test results agree with the general behaviour of the system
**Abnormal behaviour test**	Increases the initial investment ratio	Changes the assumptions of the model to determine any abnormal behaviour	Test results agree with the general behaviour of the system
